# Impact of COVID-19 pandemic on the availability of maternal and child health products and childhood vaccines

**DOI:** 10.1186/s40545-023-00541-4

**Published:** 2023-03-02

**Authors:** Tsegaye Melaku, Desta Assefa, Fanta Gashe, Mestawet Getachew, Temesgen Kabeta, Zeleke Mekonnen

**Affiliations:** 1 School of Pharmacy, Institute of Health, Jimma University, Jimma, Ethiopia; 2 Department of Health Policy and Management, Institute of Health, Jimma University, Jimma, Ethiopia; 3 School of Medical Laboratory Sciences, Institute of Health, Jimma University, Jimma, Ethiopia

**Keywords:** Availability, Childhood vaccines, COVID-19, Maternal and child health, Stock status

## Abstract

**Background:**

In many low- and middle-income countries, the 2019 novel coronavirus (COVID-19) has challenged efforts to ensure access to and availability of quality maternal, newborn, and child health (MCH) services and essential MCH commodities.

**Objectives:**

This study evaluated the impact of COVID-19 pandemic on the availability of maternal and child health products and childhood vaccines at selected health facilities in Ethiopia.

**Methods:**

We have prospectively assessed 28 maternal–child health products and 14 childhood vaccines and accessories, which are listed in the Ethiopian national essential medicines list. Data were collected from 5 hospitals located in the Jimma zone of Oromia regional state in the southwestern part of Ethiopia. We extracted data on drug availability, and order fill rates for these pharmaceutical products between May 2019 and August 2020.

**Results:**

The overall mean availability of selected maternal and child health products was 43.2%. It was 52.9% (range 21.0–63.6%) prior COVID-19 and 42.6% (range 19–56.4) during COVID-19 time. The average monthly orders fill rates of hospitals for the selected products ranged from 39 to 79%. Before COVID-19 the average order fill rate was near 70% of total orders placed by the hospitals. However, immediately after the COVID-19 case notification in Ethiopia, the percentage of order filled correctly in items and quantities began decreasing.

**Conclusion:**

This study illustrates that the availability of key essential medicines for maternal and child health in the study area was low. The overall stock-out situation of MCH products has worsened during COVID-19 compared to pre-COVID-19 pandemic. None of the surveyed MCH products met the ideal availability benchmark of 80% in the public hospitals. To allow governments to guarantee these products are constantly available and affordable, a variety of policy frameworks and choices addressing inevitable epidemics should exist.

## Background

In many low- and middle-income countries (LMICs), the novel coronavirus disease-2019 (COVID-19) has challenged efforts to ensure access to and availability of quality maternal and child health (MCH) services and essential MCH commodities [1, 2]. Maintaining MCH service and ensuring the availability of commodities at health facilities remains critical because, without them, women and children may suffer, and even die, from preventable causes. However, COVID-19 has worsened existing challenges in many LMICs [3, 4]. Since the first COVID-19 outbreak, LMICs' health supply chains have been forced to respond to new demands, such as changes in patient consumption dynamics, upstream supply shocks, and the requirement to guarantee that health care professionals have access to proper personal protective equipment [5, 6].

The disruptive effects of the COVID-19 pandemic and government response measures, such as lockdowns, may further negatively influence healthcare systems, with women and children among the most vulnerable to its consequences. Pregnant women and children under the age of five face the highest risk of death immediately before, during, and after birth [7, 8]. However, ensuring the availability of a limited set of high-impact, essential MCH services and commodities can mitigate these risks and improve MCH outcomes. In the context of COVID-19, ensuring consistent availability of these commodities at health facilities is critical because decreased antenatal care outpatient visits and primary care consultations for children have been reported [9, 10], which suggests that opportunities to administer care and treatment are more limited than ever. Lack of commodity stock may further discourage MCH care-seeking behavior; a full supply of essential MCH commodities is important.

According to the official data published by World Health Organization (WHO) and United Nations International Children’s Emergency Fund (UNICEF) more than 23 million children missed out on basic childhood vaccines through routine health services in 2020, which was the highest number since 2009 and 3.7 million more than in 2019 [11, 12]. Direct and indirect effects of the pandemic on MCH services could be devastating, and jeopardize the important gains made over the last several decades [12]. Similarly, the COVID-19 pandemic is expected to have profound effects on the healthcare systems of Ethiopia. The government of Ethiopia has concerns about diminished access to maternal health products and childhood vaccines [13, 14], but little evidence exists on service provision, utilization, or availability of those essential medicines. Understanding the impact of COVID-19 pandemic on the availability of key MCH medications is critical for effectively managing supply chain disruptions in the event of a future pandemic. Access to essential health services is critical to effective pandemic response. Therefore, this study aimed to assess the knock-on effect of COVID-19 on the availability of MCH products and vaccines in southwest Ethiopia.

## Methods

### Study setting and period

**Fig. 1:**
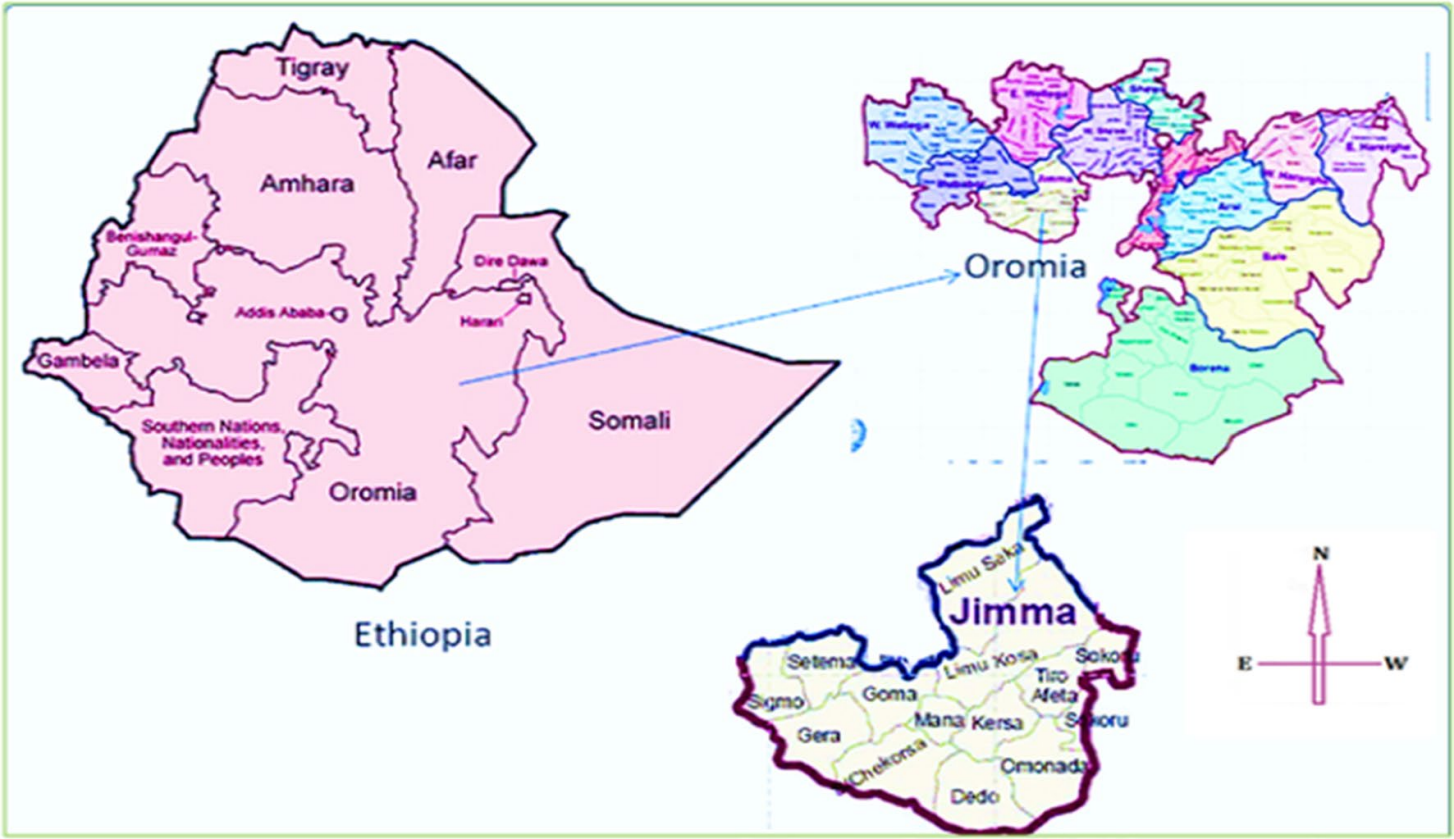
Map of Ethiopia and study area

The study was conducted at hospitals found in Jimma Zone, Oromia regional state in Ethiopia (Fig. 1). The zone has 7 hospitals (one new and the rest have been providing services for at least more than 2 years). The current study was conducted at 5 selected hospitals found in the zone. Data on stock availability, order requested and issued were extracted from the health commodity management information system of the facilities. We included 8 months pre-pandemic (May 2019–December 2019) and eight months during pandemic (January 2020–August 2020) data.

### Study design

Institutional-based cross-sectional study was employed to assess the availability of life-saving MCH products and vaccines and key challenges. In addition to quantitative approach, the study adopted the qualitative case study design, which is used as an empirical inquiry that investigates a contemporary phenomenon in depth and within its real-life context to know how COVID-19 pandemic affected the availability and stock status of MCH products.

### Selection of pharmaceutical products and measurement

According to the United Nations Population Fund (UNFPA), UNICEF, and WHO recommendations [15, 16], the commodities evaluated were among the priority medicines for mothers and children, which are used to treat primary causes of maternal death and as well as vaccine used for prevention of. The availability of MCH products and childhood vaccines was measured by using a selected supply chain management performance measuring matrix by function during the COVID-19 pandemic. This utilizes the selected indicators for each logistics activity. This assessment revealed the sum of stock levels available at the service delivery points (hospitals). Using the Logistics Management Information System (LMIS) tools one year data before the COVID-19 outbreak was compared with its status during COVID-19.

### Data source and type

The study used health facility records, pharmacy professionals working as store managers in the selected health facilities, and physical observation as data sources. Observation, physical inventory, and review of facility records, as well as DAGU (software program for managing medications) and structured interviews with health-institution pharmacy professionals, were used to collect primary data. The data included stock status, availability of MCH pharmaceutical products commodities, childhood vaccines, and the availability of various LMIS formats.

### Data collection

The investigators recruited five trained pharmacy professionals as data collectors. They received training that included the assessment’s purpose and data collection tool administration. The investigators worked closely with the data collectors and provided regular recommendations. The team visited the study facilities to check for the accuracy and completeness of the information collected.

Quantitative data were collected using a modified logistics indicator assessment tool (LIAT) developed by the USAID-Deliver Project [17] for the assessment of facility-based logistics indicators. This structured tool was used to collect data quantitatively from different source materials from health facilities stores and relevant units at the facility. And each finding was recorded for analysis.

### Data processing and analysis

Data were checked for completeness and consistency and then entered into the Statistical package for social sciences (SPSS) version 25. Descriptive statistics were used to summarize the findings and the results were presented as frequency tables and graphs. The availability of individual medicines was calculated as the percentage of sampled medicine facilities where the medicine was found. Product availability was assessed based on the WHO’s availability index A product is available if available in the health facility providing a service on the day of the visit or during the specified period. The following ranges were used for describing availability: < 30%—very low; 30–49%—low; 50–80%—fairly high; > 80%—high [18, 19].

### Ethical consideration

Before commencing data collection, ethical approval was obtained from the Institutional Review Board (IRB) of the Institute of Health, Jimma University in Ethiopia. Then, the selected hospitals were communicated with a formal letter from the health research and innovation office of Jimma University. The study was conducted in the selected health facilities after getting permission from the medical directors/managers of respective health facilities. Participants of the study were asked for written consent before participating in the study.

## Results

### Quantitative data

#### Availability of MCH products and vaccines

The stock status of MCH products and vaccines products at each hospital was measured; this included a review of stock availability for both the stock levels during COVID-19 (January 2020–August 2020) and stock levels for the 8 months before COVID-19 (May 2019–December 2019). It was calculated as:

$$\frac{{{\text{The }}\,{\text{total}}\,{\text{ products }}\,{\text{in }}\,{\text{stock}}}}{{{\text{Total }}\,{\text{number }}\,{\text{of }}\,{\text{products }}\,{\text{in }}\,{\text{the }}\,{\text{study}}}} \times 100.$$

The finding of the current study revealed that the overall mean availability of selected MCH products was 43.2%. It was 52.9% (range 21.0–63.6%, standard deviation = 13.4) before COVID-19 and 42.6% (range 19.0–56.4%, standard deviation = 9.8) during COVID-19 time. The outbreak created significant changes in the availability of MCH products in the hospitals.

The mean availability of antibiotics was fairly high before and during the outbreak of the COVID-19 pandemic. Methyldopa was fairly available before COVID-19 pandemic (56%) and its availability was decreased to 32% during the outbreak. The availability of dexamethasone 4 mg/1 ml Injection was significantly changed during COVID-19 (48%) as compared to prior the outbreak (76%). The overall mean availability of all products showed differences before and during the COVID-19 pandemic. None of the surveyed hospitals had any stocks of cefixime for the treatment of sexually transmitted diseases (Table 1).

**Table 1:** Mean availability of maternal and child health products at hospitals

S. no.	Maternal and child health products	Before COVID-19 (%)	Availability index*	During COVID-19 (%)	Availability index*
1.	Albendazole—400 mg—tablet	64	Fairly high	54	Fairly high
2.	Amoxicillin 125/250 mg dispersible tablet	66	Fairly high	52	Fairly high
3.	Ampicillin 250 or 500 mg powder for injection	76	Fairly high	64	Fairly high
4.	Azithromycin 500 mg tablet/capsule	70	Fairly high	58	Fairly high
5.	Benzathine benzylpenicillin 2.4 mU Injection	52	Fairly high	50	Fairly high
6.	Calcium-gluconate 10%/10 ml Injection	30	Low	25	Very low
7.	Chlorhexidine 21 g gel	38	Low	30	Low
8.	Cefixime 400 mg injection	0	Very low	0	Very low
9.	Dexamethasone 4 mg/1 ml Injection	76	Fairly high	48	Low
10.	Ferrous + folic acid—(60 mg + 400mcg)—tablet	70	Fairly high	64	Fairly high
11.	Gentamicin 40 mg/ml in 2 ml injection	76	Fairly high	66	Fairly high
12.	Hydralazine 20 mg injection	72	Fairly high	64	Fairly high
13.	Magnesium sulfate 50%/10 ml injection	66	Fairly high	56	Fairly high
14.	Methyldopa 250/500 mg mg tablet	56	Fairly high	32	Low
15.	Methylergometrine maleate—0.2 mg/ml—injection	52	Fairly high	38	Low
16.	Metronidazole 500 mg/100 ml infusion	52	Fairly high	40	Low
17.	Mifepristone–misoprostol (200 mg + 200mcg) tablet	52	Fairly high	44	Low
18.	Misoprostol 200 mcg tablet	36	Low	16	Very low
19.	Nifedipine (immediate) 20 mg capsule	56	Fairly high	42	Low
20.	Oral rehydration salt 20.5 g—powder	68	Fairly high	60	Fairly high
21.	Oxytocin 10 IU injection	76	Fairly high	68	Fairly high
22.	Resomal 42 g sachet	66	Fairly high	48	Low
23.	Tetanus–Diphtheria toxoid)/tetanus toxoid	72	Fairly high	66	Fairly high
24.	Tetracycline—1%—eye ointment	68	Fairly high	64	Fairly high
25.	Vitamin K1—1 mg/0.5 ml—injection	62	Fairly high	46	Low
26.	Water for injection—10 ml—injection	72	Fairly High	66	Fairly high
27.	Zinc sulphate—20 mg tablet (scored and dispersible)	72	Fairly high	64	Fairly high
28.	Zink sulphate 10 tab + oral rehydration salt 2 sachet	62	Fairly high	44	Low

#### Availability of childhood vaccines and accessories

The findings of the current study revealed that the overall availability of selected childhood vaccines and accessories was 62.3% (range 26–74%, standard deviation = 11.8) before COVID-19 pandemic. It was 54.7% (range 34–78.2%, standard deviation = 8.2) during COVID-19 pandemic. The outbreak created significant changes in the availability of childhood vaccines and accessories in the hospitals. Except for the safety box, the overall mean availability of childhood vaccines and their accessories showed differences before and during the COVID-19 pandemic (Table 2).

**Table 2:** Mean availability of childhood vaccines and accessories at hospitals

S. no.	Vaccines and accessories	Before COVID-19 (%)	Availability index	During COVID-19 (%)	Availability index
1.	BCG with diluent	70	Fairly high	66	Fairly high
2.	BOPV with droppers	64	Fairly high	54	Fairly high
3.	DPT-Hib-Hep(pentavalent)	70	Fairly high	66	Fairly high
4.	Inactivated poliovirus vaccine	64	Fairly high	56	Fairly high
5.	Measles with diluent	60	Fairly high	52	Fairly high
6.	Mixing syringe (BCG) 3 cc	75	Fairly high	64	Fairly high
7.	Mixing syringe (measles) 5 cc	74	Fairly high	66	Fairly high
8.	Pneumococcal vaccine (PCV10)	67	Fairly high	64	Fairly high
9.	Rotavirus vaccine	60	Fairly high	54	Fairly high
10.	Safety box	100	High	100	High
11.	Syringe, A-D, 0.05 ml	60	Fairly high	54	Fairly high
12.	Syringe, A-D, 0.5 ml	64	Fairly high	52	Fairly high
13.	Tetanus–diphtheria(TD)	72	Fairly high	66	Fairly high
14.	Vitamin A 100000 IU	66	Fairly high	54	Fairly high

*BCG* Bacillus Calmette–Guérin, *BOPV* bivalent oral polio vaccine, *DPT* diphtheria, pertussis, and tetanus

#### Overall orders fill rates

This indicator was used to measure the percentage of the difference between the amount ordered and the amount received for each pharmaceutical product in each month. This was calculated as:

$$\frac{{1 - \left[ {{\text{Quantity}}\,{\text{ ordered}} {-} {\text{quantity}}\,{\text{ supplied}}} \right]}}{{{\text{Quantity}}\,{\text{ ordered}}}} \times 100.$$

**Fig. 2:**
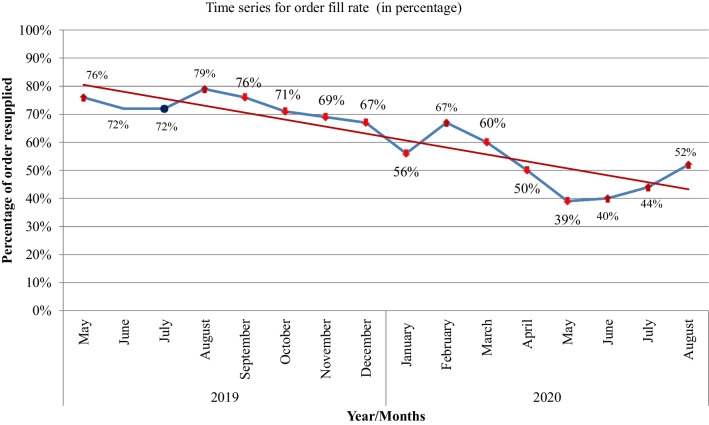
The average monthly order fill rates of hospitals

The average monthly orders fill rates of hospitals for the selected products range from 39 to 82%. Before COVID-19 the average order fill rate was near 70% of total orders placed by the hospitals. The highest (79%) order fill rate was recorded in August 2019. However, starting from the month of COVID-19 case notification, the percentage of selected medicines order(s) filled correctly in terms of items and quantities started decreasing. In March 2020, when the first COVID-19 case was reported in Ethiopia, there was a significant decrease in the order fill rate of the products at health facilities. In March 2020 only 60% of orders requested were resupplied. There was a sharp decrease in stock supply starting from March 2020 through August 2020 (Fig. 2).

## Discussion

The availability of MCH medicines makes the difference between life and death and has the biggest impact on reducing maternal mortality [15]. Hence, priority attention to ensuring commodity security should be given in countries like Ethiopia where the maternal mortality ratio remains high. Unless access is ensured, the Sustainable Development Goals (SDG) target to reduce maternal mortality with no country having over 140 per 100,000 live births by 2030 will not be achieved [20].

In the current study, the availability of MCH medicines fell short of the 80% target set by WHO. The overall mean availability of selected MCH products was 43.2%. It was slightly higher than other similar surveys conducted in east African Countries, which showed the average availability in Tanzania (37.9%), Uganda (37.9%), and Zambia (38.6%) [21]. But, it was lower when compared with the same study from Kenya (46.6%) [21] and Myanmar 52.9% [22], The results from the studies confirm the poor availability of priority medicines for women and children in the LMICs.

Across the world, the pandemic affects the supply of essential medicines used for maternal and child health. For example, similar to the report from Nepal [23], in the study area, the pandemic has made it harder to get Misoprostol (16%). Similarly, in the previous studies, the average availability of dexamethasone injection was between 84.9% and 91.4% [24, 25] in the health facilities. However, its availability significantly decreased to 48% during the pandemic. This might be associated with the rise in demand for dexamethasone after some study finds the medication can be used to manage severe cases of COVID-19 [26, 27] and global problem in the supply of essential medicines.

Relative to other MCH products antibiotics were fairly available before and during the COVID-19 pandemic. However, similar to other previous studies [28, 29] the cefixime 400 mg, the antibiotic used for the treatment of sexually transmitted diseases was completely absent in selected health facilities. This might be due to the dependence on the previous guideline and also the intention to reserve the antibiotics for other infections caused by resistance pathogens.

Before COVID-19 pandemic, oxytocin (76%) and magnesium sulphate (66%) was fairly available. This was in line with a study from sub-Saharan Africa which also reported their average availability of 81%, and 54%, respectively [30]. However, the mean availability of the two medicines decreased during the COVID-19 pandemic. This was lower than the study report from Mali [31], where the availability of magnesium sulfate (73.7%) and oxytocin (99.5%) was reported from facilities. This might be due to differences in data collection time and level of health facilities. The study from Mali also included pre-COVID-19 time and assessed data from lower primary care hospitals.

In the current study, the availability of 14 childhood vaccines and accessories was assessed. The overall mean availability of selected childhood vaccines and accessories was 62.3%. This was similar to a study from Libya [32], which reported average availability of 60%. This is indicative of plummeting rates of basic vaccinations among the world’s children. Stock management concerns, vaccine quality issues, procurement delays, worldwide vaccine shortages, financing delays, and other unspecified challenges have been mentioned as reasons for stockouts in LMICs [33]

Moreover, the pandemic worsened the shortages of autodisable (AD) syringes. For example, the average availability of Syringe (A–D) 0.5 ml was 64% before COVID-19 pandemic and decreased to 52% during the pandemic. Such shortage was reported in most of LMICs [34]. In LMICs, nearly all vaccines are administered with a 0.5-mL AD syringe. This might be associated with the increase in the rollout of COVID-19 vaccines, which increases a massive syringe shortage due to high consumption. In general, the current study showed that the mean availability of childhood vaccines and their accessories was less than 70%. This is expected to impact the SDG of universal health coverage by 2030. Therefore, achieving the bold vision laid out in the global immunization agenda 2030 [35, 36], which aims to vaccinate 90% of children and adolescents worldwide, will be possible only with strong multilateral cooperation energized by political will.

Ethiopia already faced a chronic shortage of essential neonatal health drugs and commodities [37]. The COVID-19 pandemic could worsen the shortage by disrupting procurement and importation, and transportation of essential neonatal health commodities. One of the raised issues as a problem for the shortage of the products was the financial capacity to procure commodities. Particularly, in LMICs' capacity to procure essential neonatal health commodities are compromised by economic losses from the COVID-19 pandemic, countries restricting the export of medical supplies, as well as the shrinking global development assistance from different donors [38–40]. Moreover, transportation of essential commodities through global supply chains becomes a challenge due to the travel bans and restrictions imposed across borders [41]. This is a significant lesson learned from the COVID-19 pandemic, in which geographical diversification of vaccine production is required to fulfill local demands and increase immunization uptake, particularly in LMICs.

### Limitation of the study

Despite the fact that the current study provided critical evidence on the impact of the COVID-19 pandemic on the availability of critical MCH medications and childhood vaccines, the qualitative aspects of the study were weak. One of its limitations was its small sample size which will not adequately describe the magnitude of the challenges in the supply management of life-saving MCH products and vaccines.

## Conclusion

This study illustrates that the availability of key essential medicines for maternal and child health in the study area was low. The overall stock-out situation of MCH pharmaceutical products in the study area has worsened during COVID-19 compared to pre-COVID-19 time. None of the surveyed MCH products met the ideal availability benchmark of 80% in the public hospitals. The COVID-19 pandemic continues to have broad-reaching effects on MCH and seems to be affecting the availability of MCH services and commodities. To allow governments to guarantee these products are constantly available and affordable, a variety of policy frameworks and choices addressing inevitable epidemics should exist. Creative solutions in the supply chain are needed to offer new ways to tackle COVID-19-related challenges.

## Data Availability

The datasets used and/or analyzed during the current study are available from the corresponding author on reasonable request.

## Abbreviations

COVID-19: Corona virus disease-2019
IRB: Institutional review board
LIAT: Logistics Indicator Assessment Tool
LMICs: Low- and middle-income countries
LMIS: Logistics Management Information System
MCH: Maternal and Child Health
SPSS: Statistical Package for Social Sciences
UNFPA: United Nations Population Fund
UNICEF: United Nations Children’s Fund
WHO: World Health Organization
